# RefineFuse: an end-to-end network for multi-scale refinement fusion of multi-modality images

**DOI:** 10.1007/s44267-025-00087-w

**Published:** 2025-09-24

**Authors:** Chengcheng Song, Hui Li, Tianyang Xu, Xiao-Jun Wu, Josef Kittler

**Affiliations:** 1 School of Artificial Intelligence and Computer Science, Jiangnan University, Wuxi, Jiangsu China; 2 Centre for Vision, Speech and Signal Processing (CVSSP), University of Surrey, Guildford, GU2 7XH UK

**Keywords:** Image fusion, Multi-scale interaction, Attention mechanisms, Deep learning

## Abstract

The goal of multi-modality image fusion is to integrate complementary information from different modal images to create high-quality, informative fused images. In recent years, significant advances have been made in deep learning for image fusion tasks. Nevertheless, current fusion techniques are still unable to capture more intricate details from the source images. For instance, many existing methods used for tasks such as infrared and visible image fusion are susceptible to adverse lighting conditions. To enhance the ability of fusion networks to preserve detailed information in complex scenes, we propose RefineFuse, a multi-scale interaction network for multi-modal image fusion tasks. To balance and exploit local detailed features and global semantic information during the fusion process, we utilize specific modules to model cross-modal feature coupling in both the pixel and semantic domains. Specifically, a dual attention-based feature interaction module is introduced to integrate detailed information from both modalities for extracting shallow features. To obtain deep semantic information, we adopt a global attention mechanism for cross-modal feature interaction. Additionally, to bridge the gap between deep semantic information and shallow detailed information, we gradually incorporate deep semantic information to shallow detailed information via specific feature interaction modules. Extensive comparative and generalization experiments demonstrate that RefineFuse achieves high-quality fusions of infrared, visible, and medical images, while also facilitating advanced visual tasks, such as object detection.

## Introduction

Due to the divergent imaging principles underlying standard imaging devices [1], it is challenging to obtain all scene information using a single device [2, 3]. In the context of multi-modal sensing, image fusion becomes a crucial technique used to extract meaningful information from different source images and aggregate it into a fused image [4, 5]. Image fusion techniques have the capacity to leverage the complementary strengths inherent to each individual modality and compensate for the weaknesses of individual modalities [6]. The fusion techniques have applications in a variety of fields, including medical image fusion [7, 8], multi-focus image fusion [9], multi-exposure image fusion [10–12], remote sensing image fusion [13, 14] and other applications [11, 15, 16].

In the domain of image fusion, infrared-visible image fusion (IVF) and medical image fusion (MIF) are widely recognised for their practicality. Visible images offer a natural perception and high resolution; however, they are susceptible to variations in light conditions. Although infrared images typically capture salient information, they have lower resolution and do not provide colour information. The fused images usually contain more information about the scene and can improve the performance of subsequent high-level vision tasks, such as semantic segmentation [17, 18], object tracking [19, 20] and object detection [21]. Similarly, in medical imaging, computed tomography (CT) primarily describes extremely dense tissues (such as bones), while magnetic resonance imaging (MRI) primarily provides structural information about soft tissues [22]. Positron emission tomography (PET) and single-photon emission computed tomography (SPECT), among other imaging modalities, reflect information such as metabolic activity and blood flow in different tissues [23]. The primary objective of MIF is to integrate images acquired from various imaging techniques to provide a clear composite representation, thereby aiding in diagnosis and treatment [24].

Non-deep image fusion methods are usually performed in the spatial and transform domains [25]. Representative methods include the discrete wavelet transform (DWT) [26], sparse representation (SR) [27, 28], low-rank representation (LRR) [29, 30] and multi-scale transform [31–33].

In recent years, with the continuous development of deep learning, researchers have been enhancing fusion performance by combining methods or architectures such as convolutional neural network (CNN) [1], Transformer, generative adversarial network (GAN) [34], diffusion model [35], and auto-encoder(AE) [36].

**Figure 1:**
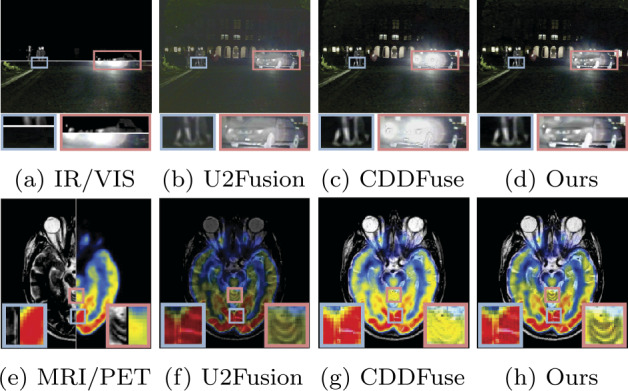
A comparison of different methods. Our method performs in complex scenarios, such as overexposed scenes, for infrared and visible image fusion tasks, as well as in medical image fusion tasks. (a) Infrared (IR)/ visible (VIS) images. (b) IR/VIS image fusion results provided by Ref. [37]. (c) IR/VIS image fusion results provided by Ref. [38]. (d) IR/VIS image fusion results using our method, RefineFuse. (e) Medical images. (f) Medical image fusion results provided by Ref. [37]. (g) Medical image fusion results provided by Ref. [38]. (h) Medical image fusion results using our method, RefineFuse

Although the aforementioned methods have achieved satisfactory fusion results in most scenarios, there are still inadequacies in the fusion results in complex environments, such as the influence of strong light at night. In each of the datasets for the two tasks, we have selected a typical example, as shown in Fig. 1. Due to the susceptibility of visible sensors to lighting conditions, pedestrians and vehicles appear submerged in darkness and intense light, respectively, in the infrared and visible images. Although the fusion result using U2Fusion includes detailed information from the visible image and salient information from the infrared image, both scene and salient information are noticeably weakened. While CDDFuse retains pedestrian information from the infrared image, much of the vehicle information is obscured by intense light. Similarly, in medical image fusion, the fusion result using U2Fusion significantly weakens the information of the source images, while the fusion result using CDDFuse lacks the ability to preserve detailed information.

Inspired by relevant studies adopting multi-scale network architectures [39, 40] and motivated by the limitations of existing methods, this paper proposes a multi-modal image fusion network with a multi-scale interaction mechanism, named RefineFuse. In RefineFuse, multi-scale features are first extracted from the source images to balance and fully exploit local detailed information and global semantic information. Feature interaction is achieved using specific modules. Specifically, shallow features contain more detailed information, whereas deep features contain more semantic information. Here, we utilize the superficial feature interaction module (SFIM) to handle shallow features. Moreover, to enhance the contextual awareness of the fusion network, cross-attention mechanism is introduced into the profound semantic interaction module to handle the interaction of deep features. Finally, the combination of specific shallow and deep feature interaction modules, along with a set of decoders, produces the final fusion result. Due to this architectural design, our fusion results are richer in detail while preserving salient information from the source images. Figure 1 presents a visual comparison of the fusion results produced by our method (RefineFuse) and two representative image fusion approaches. In summary, the contributions of this paper are as follows:

1. A new multi-modal image fusion method is proposed, which leverages different feature interaction strategies for shallow and deep features to fully exploit local detailed information and global semantic information.
2. A superficial feature interaction block (SFIM) based on dual attention and a deep-superficial feature interaction block (FIM) are proposed. SFIM is designed to enable meaningful interactions between features from different modalities to preserve detailed information, while FIM aims to bridge the gap between features of different depths.
3. Compared to existing state-of-the-art, our method exhibits superior fusion performance in terms of both visual effects and quantitative metrics. We also show that our method enhances the performance of the downstream object detection task.

The structure of the paper is given as follows. In Sect. 2 we introduce the techniques related to the approach proposed in this paper. In Sect. 3 we describe our network structure and the fusion network in detail. In Sect. 4, we present the qualitative and quantitative results obtained on different benchmarking datasets and by applications in downstream object detection task to demonstrate the merits of the proposed approach. The results of the ablation studies validate our design choices. Further supporting evidence for our approach is provided by the subjective evaluation of the fusion results. Finally, concluding remarks are given in Sect. 5.

## Related work

In this section, we briefly reviewed some techniques relevant to our method, including deep learning-based multi-modal image fusion, and attention mechanisms.

### Deep learning-based methods

The autoencoder-based method is a two-stage image fusion method. It is trained on large natural image datasets (e.g. MS-COCO [41] dataset and ImageNet [42] dataset). It is then deployed for extracting features for the two modalities. A manually designed fusion strategy is used to fuse the deep extracted features. The fused image is reconstructed using the fused features and a decoder. Examples include DenseFuse [36] and NestFuse [43]. As a manually designed fusion strategy is unable to assign appropriate weights to the deep features, which limits the fusion performance, RFN-Nest [39] proposes a learnable fusion strategy. However, the fusion result may still be compromised by the performance of the auto-encoder, which is not necessarily tuned for infrared images. Many end-to-end image fusion methods have been proposed, such as FusionGAN [34], SeAFusion [44], U2Fusion [37] and SwinFusion [22]. These methods differ in terms of network structures and loss functions to achieve feature extraction, feature fusion and image reconstruction. In 2019, Ma et al. [34] for the first time intruduced the GAN method into the image fusion task, where the discriminator forces the generator to produce fused images with more information. Although the method achieves good fusion results, it fails to preserve texture detail. This motivated the introduction of loss functions such as detail loss and texture loss to improve the fusion performance. However, the training process of the GAN-based method is complicated and the generator cannot control the details well enough.

In 2020, Xu et al. [37] designed U2Fusion, a fusion network applied to multiple fusion tasks. U2Fusion achieves good results in most fusion tasks, but the fused images were not visually appealing. In 2022, Tang et al. [44] proposed SeAFusion, which combines the fusion task with downstream vision tasks (e.g. object detection and semantic segmentation) to drive the network to retain as much semantic information as possible by introducing a novel loss function.

Recently, some contrastive learning-based fusion frameworks such as CONAN [45] and CLOVEN [46], originally proposed for multi-view representation learning, have demonstrated that explicitly modeling both consistent and complementary information between modalities can enhance the robustness and generalization of fused representations. MODfinity [47] dynamically controls multi-modal information flow through modal affinity, effectively mitigating error propagation caused by uneven information quality across different modalities. It guides information interaction at both feature and label levels, enhancing the robustness of multi-modal systems in noisy environments. Although originally designed for clustering, classification, and domain adaptation tasks, their core principles offer valuable insights for multi-modal image fusion, particularly in the design of fusion modules and alignment objectives.

Furthermore, in 2022, Ma et al. [22] applied the network structure of Swin-Transformer [48] to image fusion, called SwinFusion, which is designed to achieve multiple image fusion tasks using a unified network. However, it is more computationally intensive. The RefineFuse fusion network proposed in this paper provides a more sophisticated fusion of features by fusing them at different scales. Our approach achieves better fusion results at lower computational costs.

### Attention mechanisms

The attention mechanism is a widely used technique in computer vision tasks, inspired by research on human vision. In cognitive science, humans often selectively focus on a portion of all information, a mechanism commonly referred to as attention [49]. In recent years, the attention mechanism has been extensively applied in computer vision tasks such as image enhancement, semantic segmentation, object detection, and object tracking.

In the CNN, spatial and channel attention are common attention mechanisms. Due to their practicality, researchers often combine these two attention methods. For example, in 2016, Chen et al. [50] proposed spatial and channel attention, known as SCA-CNN. They applied this combined attention to image captioning tasks and achieved excellent results. Additionally, Liu et al. [51] introduced edge attention mechanisms into the fusion of infrared and visible images to enhance the network’s ability to focus on texture details. In our study, spatial attention is employed to handle shallow-level detail features. Parallel branches, composed of max-pooling and average-pooling operations, are utilized to process features from different modalities, extracting more effective information for feature interaction. In feature interaction at varying depths, a combination of channel attention and spatial attention is employed to compensate for differences in features at different levels.

The self-attention mechanism is an improvement on attention mechanisms, focusing on internal information to reduce reliance on external information. For example, Vaswani et al. [49] first utilized the self-attention mechanism in sequence models, replacing recurrent neural networks, in a network architecture called Transformer. Subsequently, due to the Transformer’s outstanding long-range modeling capabilities, it has been widely used in computer vision tasks. Many Transformer-based models have achieved excellent results in various visual tasks such as image fusion, semantic segmentation, and object tracking. Considering the significant computational cost of self-attention in Transformers, many Transformer variants have been proposed. For instance, Restormer [52] improves Transformer blocks in image restoration tasks by incorporating multiple Dconv heads and transpose attention, achieving remarkable results. In our work, we introduce similar operations in deep features to enable fusion networks to focus on global contextual information.

## Approach

**Figure 2:**
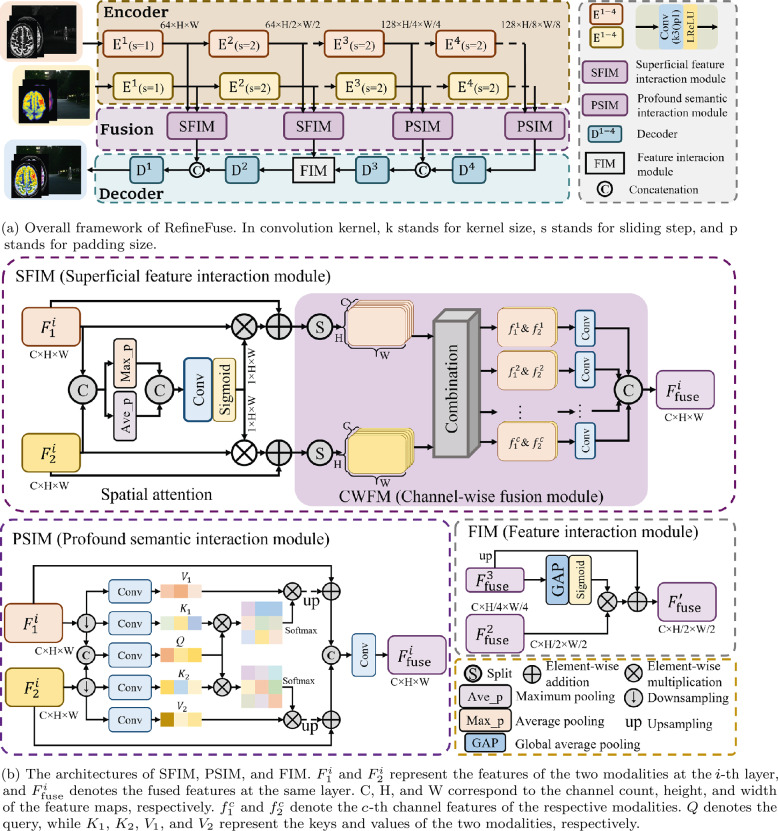
The overall framework of RefineFuse and the architecture of each module

In this section, we will provide a detailed introduction to our multi-modal image fusion method, namely RefineFuse. The detailed structure of RefineFuse is shown in Fig. 2. Initially, we extract multi-layered pyramid-like features of different scales from the source images through the encoder. These extracted features of various scales are then fed into corresponding feature interaction modules for fusion, and finally reconstructed by the decoder to obtain the ultimate fused image.

### Overview of the proposed method

For the given aligned images of different modalities, denoted as $I_{1}\in \mathbb{R}^{H\times W\times 1}$ and $I_{2}\in \mathbb{R}^{H\times W\times 3}$, and their fused image $I_{\mathrm{f}}\in \mathbb{R}^{H\times W\times 1}$, RefineFuse consists mainly of three parts: feature extraction, fusion layer, and image reconstruction. Specifically, in the feature extraction stage, $I_{1}\in \mathbb{R}^{H\times W\times 1}$ and $I_{2}\in \mathbb{R}^{H\times W\times 3}$ are fed into two separate encoding paths to extract features $F_{1}^{i}$ and $F_{2}^{i}$ at different scales, where *i* represents different levels. Each modality’s encoding path consists of four convolutional layers with kernel sizes of 3 × 3. The first convolutional layer has a stride of 1, while the rest have a stride of 2. Then, based on the levels of $F_{1}$ and $F_{2}$, they are fed into specific feature interaction modules.

In our design, since shallow features contain more texture details, we use the superficial feature interaction module (SFIM) with spatial attention to achieve interaction between shallow features of the two modalities. For deep features, to enhance the contextual awareness of information from different modalities, spatial self-attention mechanism is introduced into the profound semantic interaction module (PSIM) to achieve interaction between deep features of the two modalities. The feature reconstruction path consists of four encoders and a feature interaction module, mainly responsible for reconstructing the fused image from multi-scale fusion features. The design of the feature interaction module (FIM) is mainly used to bridge the gap between different levels of features. In the decoding path, the first decoder consists of four convolutional layers, with the activation function of the last convolutional layer being Tanh, and the rest being ReLU. The remaining three decoders consist of a 1 × 1 convolution, a 3 × 3 convolution, and bilinear interpolation operations for upsampling features.

#### The structure of SFIM

Due to the differences between images of different modalities, meaningful information is often distributed in different spatial regions [37, 53], which brings challenges to accurate feature alignment and fusion. In order to fuse the key features of different modalities, we first use spatial attention to generate spatial attention maps for different modalities, thereby enhancing meaningful information in each modality and suppressing redundant or useless information. Then, we use a per-channel fine fusion method to complete the interaction of modality-specific details. The details of SFIM are shown in Fig. 2(b). Specifically, we first concatenate the two modalities in the channel dimension, and then apply pooling operations composed of max pooling and average pooling to the concatenated features in the channel dimension, compressing the concatenated features into two feature maps ($F_{\mathrm{max}}$, $F_{\mathrm{avg}}$). Afterwards, the feature maps are processed into two-channel spatial attention maps by a convolutional layer consisting of a 3 × 3 convolution and a Sigmoid. Finally, the enhanced features are obtained through the following calculation:

1 $$ \begin{aligned} \hat{F}_{1}^{i} = F_{1}^{i}\oplus (F_{1}^{i}\otimes \delta (Conv( \mathcal{C}(F_{\mathrm{max}}, F_{\mathrm{avg}}))), \\ \hat{F}_{2}^{i} = F_{2}^{i}\oplus (F_{2}^{i}\otimes \delta (Conv( \mathcal{C}(F_{\mathrm{max}}, F_{\mathrm{avg}}))), \end{aligned} $$

where ⊕ refers to element-wise summation, ⊗ indicates elementwise multiplication, $\mathcal{C}$ represents the concatenation operation in the channel dimension, and *δ* and $Conv$ denote the sigmoid function and a convlution layer, respectively. Subsequently, inspired by the fine-grained feature grouping strategy [54, 55], we first group the feature maps of the two modalities along the channel dimension, and then employ a per-channel feature interaction strategy for local detail information fusion, thereby enabling effective modality-specific feature integration while reducing cross-channel interference. Finally, the aggregated features are concatenated along the channel dimension to obtain the fused feature $F_{\mathrm{fuse}}$. Specifically, $\hat{F}_{1}$ and $\hat{F}_{2}$ are grouped in channel dimension to obtain $f_{1}^{a}$ and $f_{2}^{a}$, respectively, where *a* ∈ {1, 2, …, *c*}. The feature maps of the two modes with the same location are spliced, and then the spliced feature maps are connected in the channel dimension, and refined and fused by 3 × 3 grouped convolution to obtain the final fused feature map $F_{\mathrm{f}}$. The specific process can be formulated as follows:

2 $$ \begin{aligned} f_{1\&2}^{a} &= f_{1}^{a}\&f_{2}^{a} = \mathcal{C}(f_{1}^{a}, f_{2}^{a}), \\ f^{a} &= Conv^{i}(f_{1\&2}^{a}), \\ F_{\mathrm{fuse}} &= \mathcal{C}(f^{1},f^{2},\ldots,f^{c}), \end{aligned} $$

where $Conv^{i}$ denotes the 3 × 3 convolution and $\mathcal{C}(\cdot )$ denotes the concatenation operation, which are all performed in the channel dimension.

Compared with the simple splicing method in the channel dimension [44], the group splicing method can fuse the features of the two modalities in a finer way and avoid the interference of the useless information of other channels, which is conducive to the subsequent recovery of the information and the preservation of the detailed information, so as to achieve better fusion effect of the network.

#### The structure of PSIM

Given the semantic differences and correlations of deep-level features between the two modalities, we have designed a profound semantic interaction module (PSIM), inspired by Tang et al. [56] and based on cross-attention, to handle deep-level features. By facilitating global interaction of deep semantic features, we aim to model the semantic relationships between the two modalities globally, enabling the network to learn the interdependencies and relations between them. The detailed structure of PSIM is illustrated in Fig. 2(b). Due to the exponential relationship between the computational complexity of the self-attention mechanism and the input size, larger inputs result in increased computation time. To reduce computational cost, the features in the third and fourth layers of the network are downsampled to 1/4 and 1/8 of the original resolution, respectively. PSIM initially processes the downsampled features through a projection function composed of convolution and reshaping operations to obtain keys $K_{x}^{i}\in \mathbb{R}^{H_{i}W_{i} \times C_{i}}$ and values $V_{x}^{i}\in \mathbb{R}^{H_{i}W_{i} \times C_{i}}$ for both modalities. We merge the features of the two modalities, and then use a projection function composed of convolution and reshaping operations to obtain queries $Q^{i}\in \mathbb{R}^{H_{i}W_{i} \times C_{i}}$ containing complementary attributes of the two modalities. Through this operation, we can fully utilize the complementary attributes of different modalities. The specific process is detailed as follows:

3 $$ \begin{aligned} V_{x}^{i} &= Reshape(Conv_{v}^{x}(\downarrow F_{x}^{i})), \\ K_{x}^{i} &= Reshape(Conv_{k}^{x}(\downarrow F_{x}^{i})), \\ Q^{i} &= Reshape(Conv_{q}^{x}(\mathcal{C}(\downarrow F_{1}^{i}, \downarrow F_{2}^{i}))), \end{aligned} $$

where $x\in \{1, 2\}$ denotes the modality, $Conv(\cdot )$ represents a convolutional layer with a 3 × 3 kernel size, $Reshape$ denotes a reshaping operation, *H*, *W* and *C* represent the height, width, and number of channels of the features, respectively, and $\mathcal{C}(\cdot )$ denotes concatenation operation. Then, we calculate the semantic relationships for each modality using Eq. (4) to obtain the global attention maps $A_{x}^{i}\in \mathbb{R}^{H_{i}W_{i}\times H_{i}W_{i}}$ for different modalities:

4 $$ \begin{aligned} A_{x}^{i} &= Softmax(Q^{i}{K_{x}^{i}}^{\text{T}}) \end{aligned} . $$

Next, we multiply the values of different modalities by their respective attentions to obtain features with global context. Then, we upsample the global features using bilinear interpolation to the same size as the input and add them to the original features. Finally, we concatenate the features along the channel dimension and input them into a 3 × 3 convolutional layer to obtain the fused features $F_{\mathrm{fuse}}^{i}\in \mathbb{R}^{C_{i}\times H_{i} \times W_{i}}$.

#### The structure of FIM

Due to the rich detail and structural information contained in shallow features, while deep features mainly encompass global semantic information, there exists a disparity between deep and shallow features. Hence, a feature interaction module (FIM) is designed to bridge this gap between different layers of features. In our setup, unlike previous methods that simply concatenate features along the channel dimension, we utilize deep global semantic information to guide and enhance shallow features both in terms of channel and spatial dimensions. We introduce channel attention and spatial attention into FIM, allowing for an enhanced quality of reconstructed results from different perspectives.

Specifically, deep semantic features and shallow features are separately input into channel attention blocks composed of global average pooling and sigmoid functions to generate channel attention weights. These weights are then used to weight the shallow features. Inspired by prior works [57], we upsample the deep semantic features using bilinear interpolation and integrate them with enhanced shallow features, allowing for multi-level semantic enrichment. By doing so, we can fully leverage both local detail and global information from the source images, thereby enhancing the quality of the reconstructed fusion image. The detailed process is as follows:

5 $$ \begin{aligned} {F}_{\mathrm{fuse}}^{\prime } &= F_{\mathrm{fuse}}^{3} \oplus (F_{ \mathrm{fuse}}^{2}\otimes \delta (GAP(F_{\mathrm{fuse}}^{3}))), \end{aligned} $$

where $F_{\mathrm{fuse}}^{3}$ represents the output of the Decoder $D^{3}$, $F_{\mathrm{fuse}}^{2}$ represents the fusion result of the second layer features, which is the output of the second SFIM, $GAP(\cdot )$ represents global average pooling, ⊕ denotes element-wise addition, and ⊗ denotes element-wise multiplication.

### Loss function

To unify the modeling of multi-modal image fusion, we guide the fusion network to retain the structure, texture details, and control the pixel intensity of the source images by constraining the difference between the fused result and the source images. Here, we introduce the texture loss $\mathcal{L}_{\mathrm{text}}$ and the intensity loss $\mathcal{L}_{\mathrm{int}}$ [56] to constrain the quality of the fusion result.

We use the intensity loss $\mathcal{L}_{\mathrm{int}}$ to constrain the pixel intensity of the fusion result to remain consistent with the source images. To mitigate the potential impact of overexposed regions in the visible image on the fusion result, we combine the fusion rules of salient object masks and contrast masks. The salient object mask is obtained from semantic segmentation labels, where we set the mask value of salient object regions in the infrared image to 1. The contrast mask is obtained by computing the squared difference between pixel values and the average of surrounding pixels in the source images. Similarly, we set the mask value to 1 for positions with high contrast in the infrared image. Next, we add these two mask values together, setting the value to 1 at positions where the sum is greater than 0, thus obtaining the mask *M* for the infrared image. The masks for the infrared and visible images can be expressed as

6 $$ M_{\mathrm{ir}}=\left \{ \begin{aligned} &1,~if~M(i, j) > 0, \\ &0,~otherwise, \end{aligned} \right . $$

where *M* represents the sum of masks for salient regions and high-contrast regions in the infrared image. After obtaining the $M_{\mathrm{ir}}$ for the infrared image, it is straightforward to derive the $M_{\mathrm{vi}}$ for the visible image, where the mask value is 1 for non-salient regions and high-contrast regions in the visible image, and 0 otherwise. The intensity loss $\mathcal{L}_{\mathrm{int}}$ can be expressed as

7 $$ \begin{aligned} \mathcal{L}_{\mathrm{int}}= \frac{1}{HW}(\left \| M_{\mathrm{ir}} \otimes (I_{\mathrm{f}}-I_{\mathrm{ir}})\right \|_{1}+\left \| M_{ \mathrm{vi}}\otimes (I_{\mathrm{f}}-I_{\mathrm{vi}})\right \|_{1}), \end{aligned} $$

where $\left \|\cdot \right \|_{1}$ stands for the $l_{1}$-norm.

Additionally, to constrain the fusion network to preserve texture details and structural information from the source images, we introduce the texture loss $\mathcal{L}_{\mathrm{text}}$. The texture loss is defined as follows:

8 $$ \mathcal{L}_{\mathrm{text}}=\frac{1}{HW}\left \||\nabla I_{\mathrm{f}}|- \max (|\nabla I_{\mathrm{ir}}|, |\nabla I_{\mathrm{vi}}|)\right \|_{1}, $$

where ∇ is the Sobel operator and $\left |\cdot \right |$ represents the absolute value calculation, and $max(\cdot )$ stands for the element-wise maximum selection.

During the training phase, the overall loss can be formulated as

9 $$ \mathcal{L}_{\mathrm{total}}=\mathcal{L}_{\mathrm{text}}+\lambda \mathcal{L}_{\mathrm{int}}. $$

## Experimental validation

In this section, we first introduce the experimental details, including training and testing datasets, experimental configurations, and implementation details. Subsequently, we demonstrate the effectiveness of specific designs through a series of ablation experiments. Qualitative and quantitative comparisons are conducted with ten state-of-the-art fusion methods to showcase the superiority of our approach. Additionally, extended experiments on advanced visual tasks are conducted to validate the potential of our method in other visual tasks.

### Experimental settings

#### Datasets and metrics

We validate our RefineFuse in both infrared and visible image fusion tasks and medical image fusion tasks. For the infrared and visible image fusion task, we utilize three popular datasets: MSRS [58], M^3^FD [59], and TNO [60]. The MSRS dataset contains complex road scenes captured during both day and night, with 1083 pairs of training images and 361 pairs of testing images. The M^3^FD dataset consists of various scenarios, including strong light, low light, and smoke occlusion. Additionally, M^3^FD includes object detection labels for six categories. We conduct experiments on object detection using the M^3^FD dataset to validate the potential of our method in advanced visual tasks. The TNO dataset primarily describes military scenarios. We train our network on the MSRS training set (1083 pairs) and evaluate its performance on the MSRS test set (361 pairs), M^3^FD test set (300 pairs), and TNO dataset (42 pairs) to verify the fusion performance of our method.

For the medical image fusion task, we conduct medical image fusion experiments using the Harvard Medical Dataset, which includes 20 pairs of MRI-CT, MRI-PET, and MRI-SPECT images. The native resolution of PET images is 128 × 128, while the resolution of other images is 256 × 256. We upsample PET images to 128 × 128 to maintain consistency across modalities.

We used seven quality metrics to objectively evaluate our fusion algorithm. These include: visual information fidelity (VIF) [61], average gradient (AG), the sum of the correlations of differences (SCD), Q_abf_ [62], entropy (EN) [63], spatial frequency (SF) [61] and structural similarity index measure (SSIM). Higher metrics indicate better fusion performance and higher quality of fused images. In Ref. [64], the details of these metrics are described.

VIF [61] is a metric based on the human perception. It measures the amount of information that the fused image and the source image contain about each other from a human visual perspective. Average gradient (AG) is used to measure the gradient information of the fusion image, reflecting the texture details and structural clarity of the fusion image. The sum of correlation differences (SCD) primarily measures the difference between the fusion image and the source images, thus reflecting the performance of the fusion algorithm. EN [63] is based on information theory to calculate the amount of information contained in the fused image. Q_abf_ [62] is used to measure the edge information of the image. SF [61] measures the distribution of the gradient by measuring the gradient distribution of the fused image, which reflects texture detail information. SSIM is an index that measures the similarity between a fused image and the source image in terms of luminance, contrast, and structural information, and it effectively simulates human visual perception.

#### Implement details

Our network was implemented on an NVIDIA 2080Ti using PyTorch as the programming environment. We conducted training on the MSRS dataset. During training, images in the training set were randomly cropped into patches of 256 × 256 resolution and normalized to [0, 1]. We applied augmentation techniques such as random rotation and random flipping to the training data to enhance the model’s generalization performance. Our initial learning rate was set to 1 × 10^−3^, and the batch size was set to 8. We trained the network for 300 epochs using the SGD optimizer. In Eq. (9), the parameter *λ* was set to 5, which was used to balance the importance of intensity loss ($\mathcal{L}_{\mathrm{int}}$) and texture loss ($\mathcal{L}_{\mathrm{text}}$).

For the RGB image, it is first converted to YCbCr space. Then, the Y (luminance) channel of the RGB image is fed to the fusion network as the input because texture information and intensity information of the visible image are concentrated in this channel. Finally, the fused Y is combined with the Cb and Cr channels, which can be converted to an RGB image.

### Infrared and visible image fusion

In this section, we test our network with three datasets and select ten state-of-the-art and efficient fusion networks for comparative evaluation. These include one traditional image fusion method, MST [65], and nine deep learning-based fusion networks: DenseFuse [36], U2Fusion [37], RFN-Nest [39], SDNet [66], SeAFusion [44], SwinFusion [22], LRRNet [15], CDDFuse [38] and EMMA [67].

#### Qualitative results

**Figure 3:**
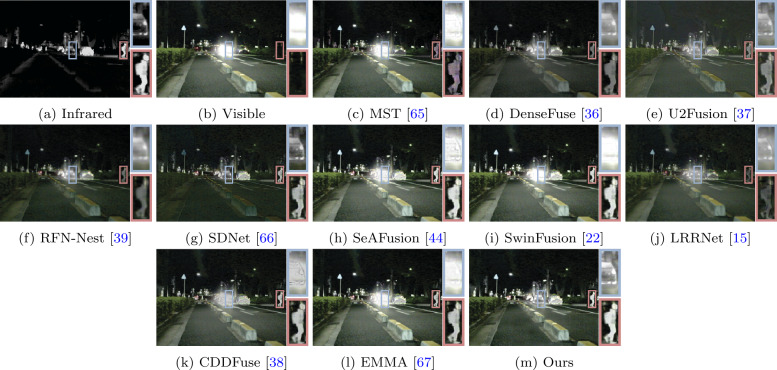
A visual comparison of our method with ten state-of-the-art methods on a typical image pairs from the MSRS [58] dataset

**Figure 4:**
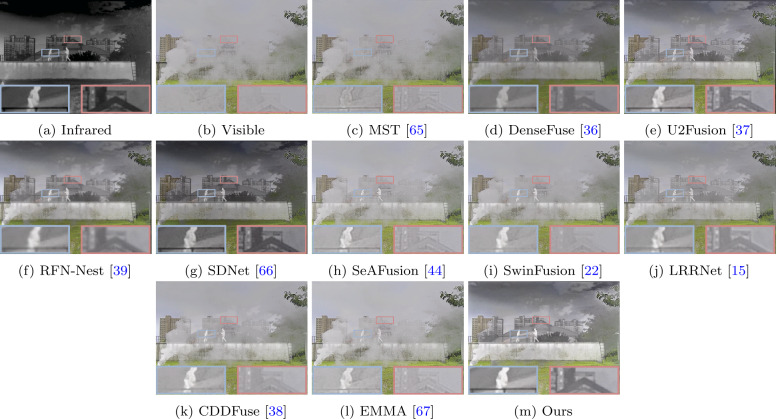
A visual comparison of our method with ten state-of-the-art methods on a typical image pairs from the M^3^FD [59] dataset

**Figure 5:**
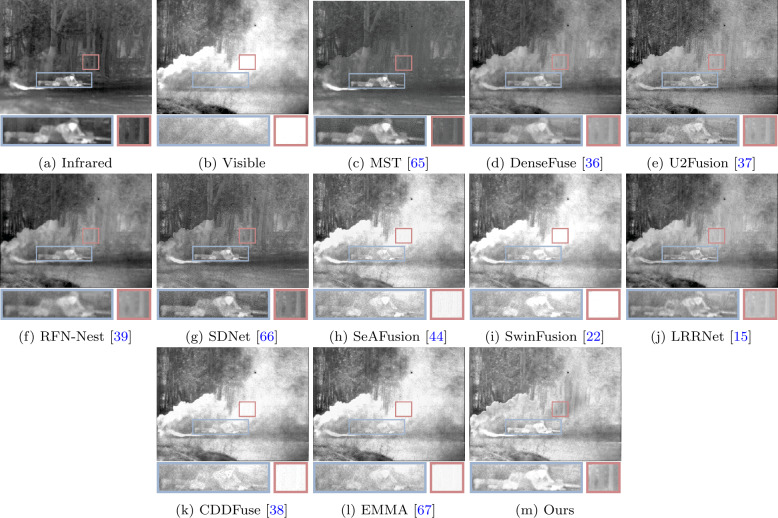
Visual comparison of our method with ten state-of-the-art methods on a typical image pairs from the TNO [60] dataset

We conducted comparative experiments on the MSRS [58], M^3^FD [59], and TNO [60] datasets to evaluate our method. The fusion results from different methods are shown in Figs. 3, 4 and 5. From Fig. 3, it can be observed that due to strong light from car headlights in the nighttime scenes, the visible images are overexposed, which causes the vehicle information to be completely obscured by intense light. Methods such as MST, SeAFusion, SwinFusion, CDDFuse, and EMMA fail to accurately describe the vehicle information due to the influence of the lights in the visible images. These methods fail to fully utilize useful information from the source images and their information capturing capability is easily affected by environmental factors. While other methods retain vehicle information in the fusion images, significant target information, such as pedestrian information in the red box, is noticeably weakened. Compared to other methods, our approach can fully utilize useful information from the source images, providing fusion images containing rich scene information and better visual perception.

The fusion results on the M^3^FD dataset are shown in Fig. 4. Similarly, methods like MST, SeAFusion, SwinFusion, CDDFuse, and EMMA fail to clearly describe the building information behind the smoke, as seen in the red box. While other methods retain building information behind the smoke, pedestrian information is not observed as much, especially in DenseFuse and SDNet. In contrast, our fusion results demonstrate excellent performance in retaining significant pedestrian information and detailed building information. This is mainly attributed to our multi-scale architecture design and the effective utilization of local and global information, allowing the fusion network to extract and preserve more information from the source images.

Our method also performs well on the TNO dataset, as shown in Fig. 5. From Fig. 5, it can be observed that due to smoke obscuring the view, soldiers and trees behind the smoke cannot be seen in the visible images. Some methods, influenced by the smoke, fail to retain the information of trees behind the smoke, such as SeAFusion, SwinFusion, CDDFuse, and EMMA. Similarly, their fusion results also fail to clearly describe the soldier information. Although other methods retain soldier and tree information, their fusion results (e.g., MST and SDNet) are more biased towards the infrared image, thereby overlooking the textural details in the visible light image, such as the vegetation information in the lower-left corner. In contrast, our fusion results highlight soldier information and provide a more comprehensive scene representation. Extensive experiments and analysis demonstrate the superiority of our method, even in complex scenarios.

#### Quantitative results

The quantitative comparison results on the MSRS, M^3^FD, and TNO datasets are presented in Tables 1, 2 and 3. During the testing phase, we utilized the test sets from MSRS and M^3^FD, along with 42 pairs of images from TNO, for comparison. We measured the quality of fusion results by calculating the mean values of seven metrics for different methods. The results on the MSRS dataset are shown in Table 1, where our method achieved the highest scores in three metrics and ranked second in two metrics. The best result in VIF indicates that our fusion results better align with human visual perception. The highest AG score indicates that our fusion images contain rich texture detail information, consistent with our qualitative results. The top-ranking Q_abf_ indicates that our fusion images retain important information from the source images and exhibit good visual effects. Our SCD and SF metrics ranked second, while EN ranked third. This is mainly due to the presence of overexposed areas in visible images in the MSRS dataset, where our method suppresses the overexposure, resulting in a loss between the amount of information in the fusion image and the similarity to the source image. Similarly, we also show advantages on the M^3^FD and TNO datasets. In the M^3^FD dataset, our method achieved the highest scores in four metrics, second in one, and third in one. In the TNO dataset, our method obtained the highest scores in two metrics, second in one, and third in one. This indicates that our fusion method exhibits excellent generalization performance.

**Table 1:** Quantitative comparison of fusion results on the MSRS [58] dataset. Bold: best, Underline: second best. VIF: visual information fidelity; AG: average gradient; SCD: the sum of the correlations of differences; Q_abf_: gradient-based metric; SF: spatial frequency; EN: entropy; SSIM: structural similarity index measure

Method	VIF	AG	SCD	Q_abf_	SF	EN	SSIM
MST [65]	0.9277	3.6693	1.6078	0.6486	11.3257	6.5246	1.0013
DenseFuse [36]	0.6920	2.0581	1.2511	0.3661	6.0255	5.9367	0.9011
U2Fusion [37]	0.4742	2.0945	1.0057	0.3150	6.7124	4.9533	0.6132
RFN-Nest [39]	0.6442	2.1523	1.4686	0.3822	6.2111	6.1838	0.7456
SDNet [66]	0.4984	2.6817	0.9855	0.3768	8.6715	5.2450	0.7167
SeAFusion [44]	0.9688	3.6968	**1.6853**	0.6745	11.1062	6.6515	0.9926
SwinFusion [22]	1.0035	3.5435	1.6907	0.6684	11.0615	*6.6196*	**1.0207**
LRRNet [15]	0.4725	2.1037	0.8266	0.3459	7.1152	5.9410	0.4393
CDDFuse [38]	0.9823	3.3135	1.4827	0.6768	10.2097	6.5312	1.0012
EMMA [67]	0.9744	3.7885	1.6294	0.6428	**11.5593**	**6.7229**	0.9688
RefineFuse(Ours)	**1.0554**	**3.8135**	1.6328	**0.7193**	11.4509	6.6006	0.9377

**Table 2:** Quantitative comparison of fusion results on the M^3^FD [59] dataset

Method	VIF	AG	SCD	Q_abf_	SF	EN	SSIM
MST [65]	0.7907	4.6538	1.2467	0.6264	14.0390	6.6411	0.9467
DenseFuse [36]	0.5914	2.6748	1.4886	0.3706	7.6399	6.3931	0.9283
U2Fusion [37]	0.6558	4.2654	1.6455	0.5494	11.5366	6.7806	0.9818
RFN-Nest [39]	0.5788	2.8775	1.7256	0.4028	7.7518	6.8623	0.8074
SDNet [66]	0.5787	4.7367	1.5215	0.5263	13.5981	6.8349	0.9567
SeAFusion [44]	0.7224	4.7818	1.5857	0.5988	13.9542	6.8464	0.9596
SwinFusion [22]	0.7829	4.5987	1.5614	0.6175	13.6540	6.8019	**1.0211**
LRRNet [15]	0.5682	3.6023	1.4628	0.4997	10.6891	6.4362	0.7956
CDDFuse [38]	0.7847	4.8828	1.6462	0.6089	14.7693	6.9070	0.9964
EMMA [67]	0.7690	**5.3414**	1.4942	0.5924	**15.2273**	6.9242	0.9142
RefineFuse(Ours)	**0.8376**	4.9859	**1.7941**	**0.6457**	14.6503	**7.0871**	0.9094

**Table 3:** Quantitative comparison of fusion results on the TNO [60] dataset

Method	VIF	AG	SCD	Q_abf_	SF	EN	SSIM
MST [65]	0.6068	4.0761	1.3722	0.4579	11.1203	6.6840	0.9805
DenseFuse [36]	0.6584	3.5600	1.7838	0.4463	8.9854	6.8193	1.0234
U2Fusion [37]	0.6189	**5.0233**	1.7839	0.4267	11.8638	6.9967	0.9450
RFN-Nest [39]	0.5593	2.6693	1.7843	0.3346	5.8745	6.9632	0.8145
SDNet [66]	0.5779	4.6117	1.5590	0.4298	11.6428	6.6948	0.9750
SeAFusion [44]	0.7042	4.9803	1.7281	0.4879	12.2525	7.1335	0.9623
SwinFusion [22]	0.7503	4.2113	1.7130	0.5215	10.7224	6.8909	**1.0360**
LRRNet [15]	0.5356	3.7249	1.4957	0.3443	9.5092	6.9578	0.8444
CDDFuse [38]	**0.7878**	4.6551	1.7649	0.5249	**12.3655**	7.0690	1.0110
EMMA [67]	0.7122	4.7698	1.7015	0.4648	11.5118	**7.1756**	0.9477
RefineFuse(Ours)	0.7516	4.6664	**1.7866**	**0.5344**	11.4335	7.1167	0.9544

Qualitative and quantitative results show that our fusion method can effectively preserve the texture detail and thermal radiation information, and the fused image is more natural and has a very good visual effect. This is attributed to our network architecture and specific module design. Our SFIM, DSIM, and FIM enable the network to fully utilize both local detail and global semantic information from the source images. The design of our multi-scale network structure effectively avoids the loss of detail information and aggregates information from different modalities at different scales. Qualitative and quantitative results also demonstrate that the fused images obtained by our method present superior performance in many aspects, including in terms of detail and visual effects.

### Application to object detection

**Figure 6:**
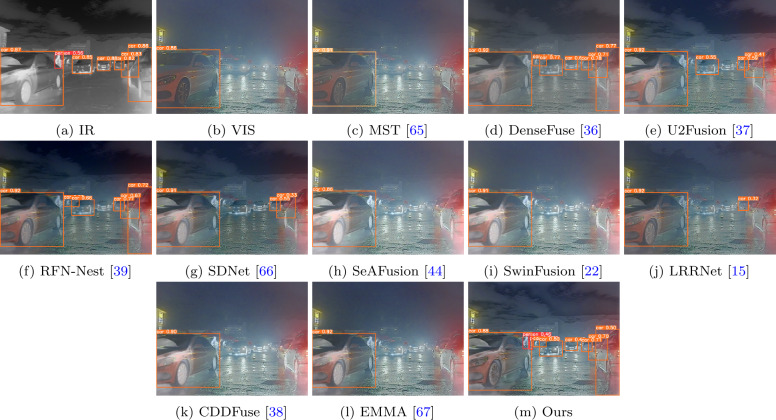
Object detection results of infrared and visible image fusion. The detector can detect more targets from our images

The task of infrared and visible image fusion is to preserve useful information from the source image and generate high-quality images with more information that can be used for advanced vision tasks. In order to verify the effectiveness of our approach for other visual tasks, we applied the fusion results to target detection. We chose YOLOv5s [68], which was pre-trained on the COCO dataset, as the object detector. For fair comparison, we conducted two sets of experiments. The first set of experiments involved directly using pre-trained YOLOv5s to detect objects in the fusion images obtained from different methods. Figure 6 illustrates the object detection results on both source images and fusion images from various methods. The second set of experiments involved retraining the detection network using both source images and fusion images from different methods. The object detection task was performed on the M^3^FD dataset [59], which comprises 4200 pairs of images. We split the M^3^FD dataset into training, validation, and test sets in an 8:1:1 ratio. YOLOv5 was trained using the SGD optimizer for 100 epochs, with a batch size of 16 and an initial learning rate of 0.01. Here, we primarily evaluate the detection performance by comparing the mean average precision (mAP) for three categories: person, car, and bus. The results are shown in Table 4.

**Table 4:** Object detection performance on the M^3^FD [59] dataset. mAP: mean average precision; mAP@0.5: mAP at IoU = 0.5; mAP@[0.5,0.95]: mAP averaged over IoU thresholds from 0.5 to 0.95 in steps of 0.05

Method	mAP@0.5				mAP@[0.5,0.95]			
	People	Car	Bus	All	People	Car	Bus	All
Infrared	81.1	89.2	75.9	82.1	48.6	61.6	34.8	48.3
Visible	72.6	91.5	90.5	84.9	39.9	65.6	48.9	51.5
MST [65]	78.6	92.0	90.1	86.9	44.6	65.8	**49.3**	53.2
DenseFuse [36]	80.1	89.8	89.4	86.4	47.6	64.8	47.9	53.4
U2Fusion [37]	81.7	91.1	88.3	87.0	49.1	66.2	46.4	53.9
RFN-Nest [39]	79.3	91.2	87.9	86.1	46.8	65.8	45.6	52.7
SDNet [66]	81.0	91.6	88.9	87.2	49.1	66.2	45.5	53.6
SeAFusion [44]	80.3	91.3	88.7	86.8	47.9	65.9	46.2	53.3
SwinFusion [22]	79.7	91.4	90.2	87.1	47.9	65.6	46.8	53.4
LRRNet [15]	79.8	91.5	**90.7**	87.3	47.6	66.0	47.6	53.7
CDDFuse [38]	80.4	91.5	88.9	86.9	47.9	66.0	47.6	53.8
EMMA [67]	79.8	91.4	89.0	86.7	47.3	65.7	47.5	53.5
RefineFuse(Ours)	**82.8**	**92.1**	90.4	**88.4**	**49.5**	**66.4**	47.3	**54.4**

The images depict the results of detection directly using pre-trained YOLOv5. From Fig. 6, it is apparent that due to the influence of lighting, the detector only detects one car in the visible image, while detecting many targets in the infrared image. Generally, fused images aggregate complementary information from source images, and the detector should detect more information from fused images. However, in real-world scenarios, due to lighting effects, the detector fails to detect all vehicle and pedestrian information from the rest of the fused images, especially in the cases of MST, SDNet, SeAFusion, SwinFusion, LRRNet, CDDFuse, and EMMA. In contrast, the detector can detect more targets, particularly pedestrian information from our fused images. This indicates that our fusion results have an advantage in texture detail.

Mean average precision (mAP) is one of the most important metrics in target detection. The mAP@0.5 indicates the result calculated when the IoU threshold is 0.5, while the mAP@0.5:0.95 indicates the result calculated when the IoU threshold varies from 0.5 to 0.95.

Table 4 displays the results of the detector trained on source images and the fused images obtained from different methods. It can be observed that the fused images, which aggregate multi-modal information, outperform single-modal object detection in terms of detection performance. Our object detection results demonstrate the best performance across different IoU thresholds, particularly in the classes of people and cars. Our overall mAP consistently ranks first. These results underscore the potential of our method in advanced visual tasks.

### Medical image fusion

The medical image fusion task was conducted on the Harvard medical dataset [69], which encompasses MRI-CT, MRI-PET, and MRI-SPECT image fusion tasks. These tasks consist of 20, 15, and 20 image pairs, respectively. We directly applied the models trained on the infrared-visible image fusion task to the medical image fusion task without fine-tuning. Similarly, we compared our method with ten previous approaches, among which U2Fusion, SDNet, SwinFusion, CDDFuse, and EMMA are multi-modal image fusion models, while the rest are designed specifically for infrared-visible image fusion. The quantitative metrics used were the same as those in the infrared-visible image fusion task.

#### Qualitative results

**Figure 7:**
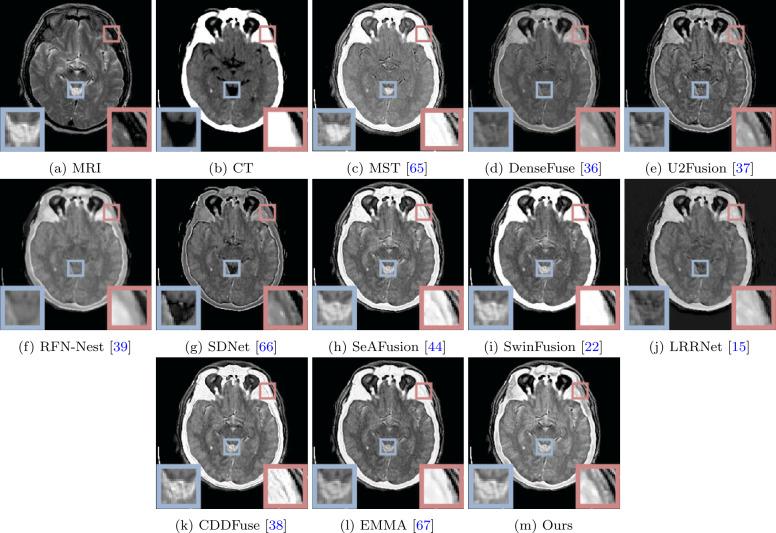
Visual comparison of our method with ten state-of-the-art methods on MRI and CT image fusion

**Figure 8:**
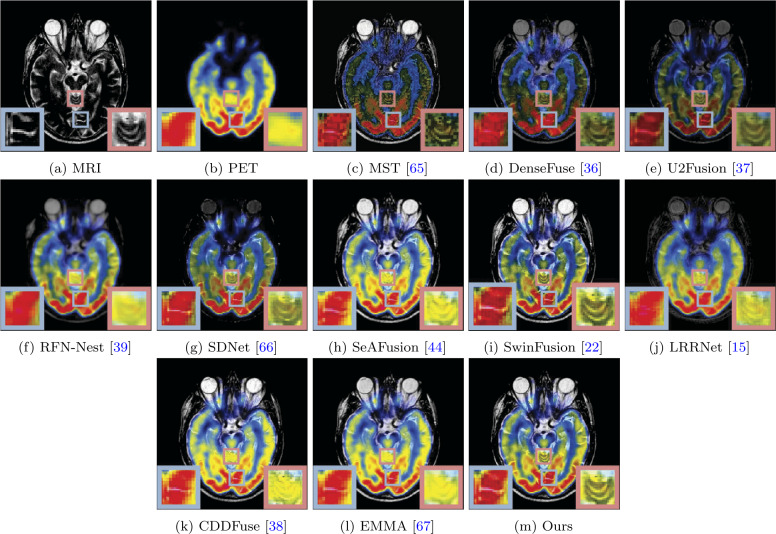
Visual comparison of our method with ten state-of-the-art methods on MRI and PET image fusion

**Figure 9:**
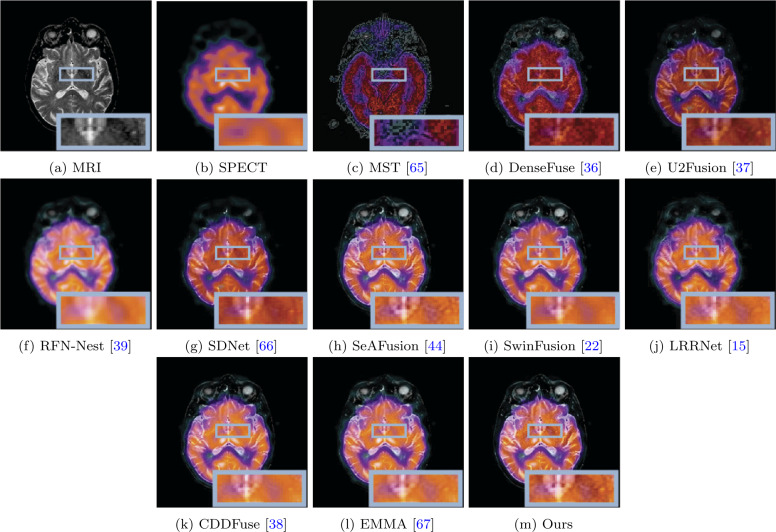
Visual comparison of our method with ten state-of-the-art methods on MRI and SPECT image fusion

The visual quality comparison of MRI-CT, MRI-PET, and MRI-SPECT are shown in Figs. 7, 8, and 9, respectively. A typical MRI-CT image fusion visualization result is illustrated in Fig. 7. In the provided image pairs, CT scans depict high-density bones (white areas) and low-density soft tissues, while MRI presents clear images of the periosteum and soft tissues surrounding the bones. It can be observed that some methods fail to preserve the white bone area of the CT images, particularly DenseFuse, U2Fusion, and SDNet. Our method not only retains the white bone area of CT scans but also preserves the clear structural information of MRI images. In comparison, MST, SeAFusion, SwinFusion, CDDFuse, and EMMA lack clear structural information in the bone area, as shown in the magnified portion within the red box. Additionally, our method effectively retains the structural information of soft tissues, as demonstrated in the area highlighted within the blue box. The image fusion visualization results of MRI-PET and MRI-SPECT are depicted in Figs. 8 and 9. While MRI delineates details of the brain, PET and SPECT primarily depict brain blood flow, oxygen, or glucose metabolism. Based on the visualization results shown in Figs. 8 and 9, most methods fail to adequately preserve the structural information in MRI images, such as MST, RFN-Nest, SeAFusion, LRRNet, CDDFuse, and EMMA. Furthermore, the fusion images produced by DenseFuse, U2Fusion, RFN-Nest, SDNet, and LRRNet significantly degrade the structural information contained in MRI images. Our results and the results of SwinFusion effectively preserve the structural information of MRI and the color information of PET and SPECT images. It is noteworthy that SwinFusion fine-tuned fusion models separately for different fusion tasks, whereas our method achieved satisfactory fusion results without fine-tuning, demonstrating its advantages and generalization performance through visual comparison.

#### Quantitative results

Tables 5, 6, and 7 present the quantitative comparison results between our method and ten other fusion approaches. In the MRI-CT fusion task, our method achieves the best performance in terms of SCD and Qabf, and ranks second in AG and SF. For the MRI-PET fusion task, our method ranks first in SCD, Qabf, and SSIM, with AG and SF ranking second. In the MRI-SPECT fusion task, our method outperforms others in VIF, SCD, and SSIM, with the remaining metrics also ranking second. The superior performance can be attributed to our method’s ability to retain complementary information from both modalities while effectively suppressing redundant or irrelevant content. Although this may slightly reduce the overall information content of the fused image compared to some methods, it enhances the representation of critical structures and details. As a result, the fusion images exhibit rich texture details, clear structural boundaries, and strong correlation with salient edges in the source images, aligning well with human visual perception. In summary, both the quantitative metrics and visual assessments confirm the superior fusion quality and robustness of the proposed method across various tasks.

**Table 5:** Quantitative comparison of fusion results from different methods on the MRI-CT fusion task

Method	VIF	AG	SCD	Q_abf_	SF	EN	SSIM
MST [65]	**0.5766**	**8.7211**	1.2052	0.5718	**38.4305**	5.0907	**0.9510**
DenseFuse [36]	0.4141	4.9992	0.9657	0.3445	19.4344	5.3632	0.6930
U2Fusion [37]	0.3351	6.4454	0.6722	0.4433	23.1866	5.3146	0.5259
RFN-Nest [39]	0.3311	4.0141	1.1025	0.2286	13.4176	5.2315	0.4307
SDNet [66]	0.3545	6.6779	0.6401	0.4567	33.7527	4.8487	0.4865
SeAFusion [44]	0.4578	8.0257	1.4731	0.5652	30.4995	5.8565	0.7561
SwinFusion [22]	0.5308	7.3610	1.5110	0.5516	30.6690	5.4823	0.8375
LRRNet [15]	0.3651	5.1767	0.1613	0.3394	22.0195	5.4706	0.3578
CDDFuse [38]	0.4379	8.0782	1.4679	0.5440	34.5958	5.1084	0.7258
EMMA [67]	0.4315	7.5324	1.3915	0.5060	27.9092	**5.9036**	0.5961
RefineFuse(Ours)	0.4561	8.2982	**1.5381**	**0.6030**	33.9496	5.5251	0.7144

**Table 6:** Quantitative comparison of fusion results from different methods on the MRI-PET fusion task

Method	VIF	AG	SCD	Q_abf_	SF	EN	SSIM
MST [65]	0.5241	**7.1950**	0.6392	0.5247	**27.3902**	3.6920	1.2954
DenseFuse [36]	**0.5294**	4.5975	0.8808	0.3504	16.3866	3.7623	1.2881
U2Fusion [37]	0.2727	3.2141	0.4350	0.1572	11.5274	3.9212	0.3051
RFN-Nest [39]	0.2270	3.1838	1.1406	0.1236	8.8637	4.4477	1.2589
SDNet [66]	0.1985	3.6697	0.8514	0.1909	17.1731	4.0759	0.2854
SeAFusion [44]	0.3348	6.3640	1.3322	0.4452	21.0883	4.7344	0.4553
SwinFusion [22]	0.3658	6.2955	1.3360	0.4475	21.8154	4.5217	0.4838
LRRNet [15]	0.0727	3.5285	0.5292	0.0989	12.7331	4.2813	0.2034
CDDFuse [38]	0.3619	6.5692	1.2690	0.4674	24.6059	4.1226	1.4468
EMMA [67]	0.2872	6.2859	1.3135	0.3783	21.1490	**5.0046**	0.4496
RefineFuse(Ours)	0.4905	7.1262	**1.4079**	**0.5552**	26.4175	4.5619	**1.4477**

**Table 7:** Quantitative comparison of fusion results from different methods on the MRI-SPECT fusion task

Method	VIF	AG	SCD	Q_abf_	SF	EN	SSIM
MST [65]	0.4787	**6.6339**	0.5142	**0.4745**	**23.5816**	3.7668	1.1181
DenseFuse [36]	0.4613	4.2949	0.8526	0.2994	14.3531	3.9010	1.1847
U2Fusion [37]	0.3398	2.9128	0.0540	0.2426	10.7121	3.8497	0.3837
RFN-Nest [39]	0.1952	2.1948	1.2368	0.0822	6.2401	4.4358	1.2409
SDNet [66]	0.2256	2.8887	0.8017	0.1820	12.3151	4.2844	0.4122
SeAFusion [44]	0.3772	4.5713	1.3077	0.3760	15.9916	4.5952	0.4776
SwinFusion [22]	0.3308	3.7229	1.2103	0.2966	13.6512	4.4417	0.5142
LRRNet [15]	0.0770	2.9480	0.4890	0.0993	10.3552	4.3713	0.2159
CDDFuse [38]	0.3325	4.4221	1.2411	0.3525	16.6733	4.1271	1.3816
EMMA [67]	0.2883	4.2077	1.2822	0.2938	15.0030	**4.9867**	0.4679
RefineFuse(Ours)	**0.5796**	5.0539	**1.3343**	0.4631	18.6618	4.5409	**1.4117**

Moreover, as shown in Tables 5-7, the MST method achieves relatively high scores on objective metrics such as AG, Qabf, and SF. However, we observed that some of its fusion results are not entirely satisfactory in terms of visual quality, as shown in Figs. 8 and 9. This discrepancy between quantitative evaluation and perceived visual performance can largely be attributed to the inherent limitations of current evaluation metrics. These metrics predominantly focus on low-level statistical characteristics, such as gradient intensity, information entropy, or pixel-level similarity, yet often fail to fully capture aspects more aligned with human visual perception, such as structural consistency, semantic saliency, and overall visual comfort. For instance, higher AG or SF values may suggest enhanced detail or richer information, but in certain cases, they may also indicate increased noise or redundant content. Additionally, attempts to preserve all information from multiple modalities can result in unnatural transitions or semantic ambiguity within the fused image. Therefore, although our method may not outperform others in all metrics, it delivers superior visual results, characterised by natural appearance, clearer structures, and better alignment with human perception.

### Ablation study

#### Ablation study for the loss function

To study the rationality of the loss function, we conducted four groups of ablation experiments. Considering that intensity loss $\mathcal{L}_{\mathrm{int}}$ is a fundamental component of the total loss function, we did not remove it in the ablation experiments; instead, we replaced it with conventional intensity loss.

*Setting A:* We replaced our mask-based intensity loss $\mathcal{L}_{\mathrm{int}}$ with conventional intensity loss $\mathcal{L}_{\mathrm{int}}$. The definition of $\mathcal{L}_{\mathrm{int}}$ is as follows:

10 $$ \begin{aligned} \mathcal{L}_{\mathrm{pixel}}=\frac{1}{HW}(\left \|I_{\mathrm{f}}-I_{\mathrm{ir}}\right \|_{1}+\left \| I_{\mathrm{f}}-I_{\mathrm{vi}}\right \|_{1}). \end{aligned} $$

*Setting B:* We replaced our mask-based intensity loss $\mathcal{L}_{\mathrm{int}}$ with intensity loss $\mathcal{L}_{\mathrm{int}}$ that selects the maximum value. The definition of $\mathcal{L}_{\mathrm{int}}$ is as follows:

11 $$ \begin{aligned} \mathcal{L}_{\mathrm{int}}=\frac{1}{HW}\left \|I_{\mathrm{f}}-max(I_{\mathrm{ir}}, I_{\mathrm{vi}}) \right \|_{1}. \end{aligned} $$

*Setting C:* To verify the impact of the $\mathcal{L}_{\mathrm{text}}$ on the fusion structure, we directly removed it.

**Figure 10:**
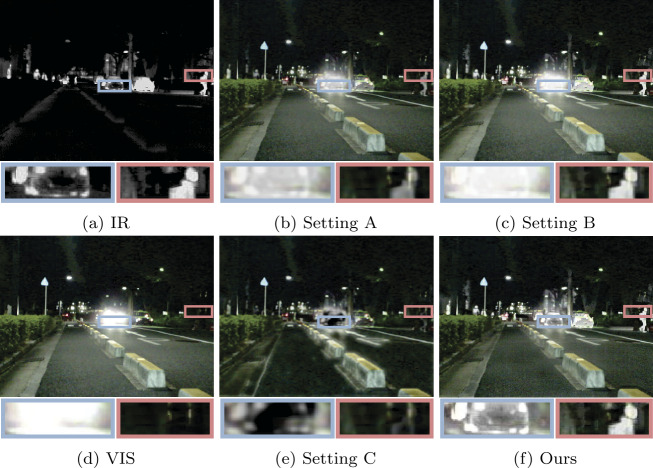
Fusion results of the ablation study on the MSRS dataset

Figure 10 shows the fusion results obtained with different settings. When using conventional intensity loss, although the results of setting A preserve vehicles under strong lighting conditions, significant pedestrians are noticeably weakened. In setting B, the fusion strategy of selecting the maximum pixel value effectively preserves salient information in the infrared image but is affected by strong lighting. In setting C, the lack of $\mathcal{L}_{\mathrm{text}}$ results in deficiencies in the texture details of the fusion image. Additionally, as seen in the quantitative results in Table 8, most metrics for the other settings show a significant decrease. These results indicate the effectiveness and rationality of our settings.

**Table 8:** Quantitative evaluation results of the ablation study

Method	VIF	AG	SCD	Q_abf_	EN	SF	SSIM
Setting A	0.8507	3.2675	1.5064	0.5639	6.4617	10.4037	**0.9771**
Setting B	0.9413	3.4603	**1.6363**	0.6196	**6.6240**	10.7415	0.9169
Setting C	0.4998	2.9337	1.0420	0.3506	6.3564	8.0533	0.5912
RefineFuse(Ours)	**1.0554**	**3.8135**	1.6328	**0.7193**	6.6006	**11.4509**	0.9377

#### Analysis of the SFIM, PSIM and FIM

**Figure 11:**
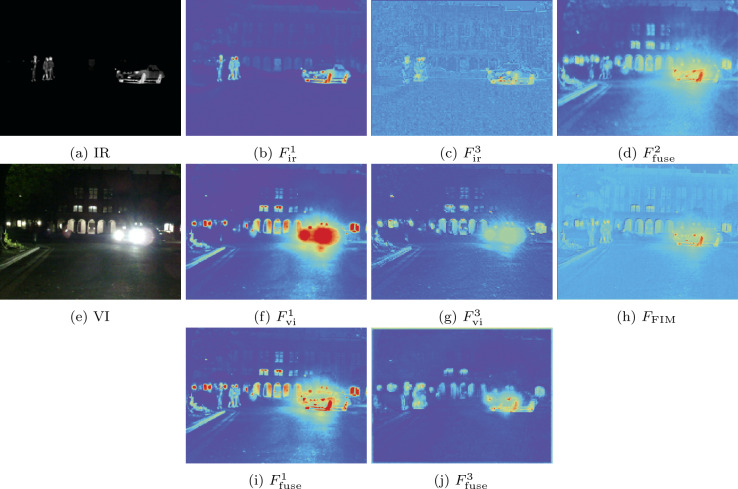
Feature visualization. $F_{x}^{i}$ represents features, where $i\in \{1, 2, 3, 4\}$ denotes the layer number, and $x\in \{\mathrm{ir, vi, fuse, FIM}\}$ denotes the features of infrared, visible, fused images, and the output of the FIM, respectively

We visualized the features from different layers of the network to demonstrate the effectiveness of our specific design. The visualization results are shown in Fig. 11. In the figure, $F_{\mathrm{ir}}^{1}$, $F_{\mathrm{vi}}^{1}$, and $F_{\mathrm{f}}^{1}$ represent the features of the first layer for infrared, visible, and fused modalities, respectively. Similarly, $F_{\mathrm{ir}}^{3}$, $F_{\mathrm{vi}}^{3}$, and $F_{\mathrm{f}}^{3}$ denote the features of the third layer for infrared, visible, and fused modalities, respectively. $F_{\mathrm{f}}^{1}$ represents the output result of SFIM, $F_{\mathrm{f}}^{3}$ represents the output result of PSIM, and $F_{\mathrm{FIM}}$ represents the output result of the FIM. From Fig. 11, it can be observed that significant pedestrian and clear vehicle information exist in the infrared features, while overexposed regions are present in the visible features, with pedestrians obscured in darkness. Upon examining the fused features, we found that our PSIM effectively preserves significant amount of pedestrian and vehicle information. From the output result $F_{\mathrm{f}}^{3}$ of the PSIM, the deeper layers of the network effectively focus on global semantic information. Through the FIM, shallow detail features and deep semantic features are interacted. As shown in Fig. 11(h), FIM effectively utilizes deep semantic information to enhance shallow features. For example, it enhances pedestrian information and suppressing strong light. Figure 11 presents that our specific design achieves the expected results. The feature visualizations above demonstrate the effectiveness of our design.

#### Ablation study for SFIM, PSIM and FIM

To validate the effectiveness of the specific design, we conducted ablation experiments on the network structure and specific modules. In Table 9, A, B, and C represent SFIM, PSIM, and FIM, respectively. Specifically, we first validated the network structure without using multiple scales, and the fusion strategy employed conventional concatenation and convolution operations. Subsequently, we verified the effectiveness of the SFIM module, which facilitated cross-modal feature interaction. The results showed a significant improvement in most metrics. Next, we designed the network with two scales, conducting experiments with and without the PSIM module to verify the effectiveness of global feature interaction. The results demonstrated considerable improvement in most metrics when global feature interaction was present. Considering network complexity and computational efficiency, we designed up to four scales at most in our experiments. Due to the deepening of the network, deeper layers affected original scale edge information because of the smaller scale. This was primarily caused by the loss of original edge information after downsampling the source image. Directly interacting smaller scale features with original scale features after simple upsampling would affect edge information. To address this, we designed the FIM to bridge the gap in features between different scales. Overall, our approach yielded promising results.

**Table 9:** Quantitative evaluation results of the ablation study

		A/B/C	VIF	AG	SCD	Q_abf_	EN	SF	SSIM
1-layer	I		1.01	3.63	1.50	0.69	6.57	11.29	0.93
	II	✓	1.00	3.77	1.54	0.65	6.61	11.19	0.92
2-layer	III	✓	1.00	3.76	1.55	0.65	6.64	11.20	0.90
	IV	✓/✓	1.02	3.82	1.52	0.72	6.59	**11.74**	0.91
4-layer	V	✓/✓	1.04	**3.84**	1.59	0.69	**6.95**	11.29	0.93
	Ours	✓/✓/✓	**1.06**	3.81	**1.63**	**0.72**	6.63	11.45	**0.94**

#### Ablation study on the number of downsampling layers

To investigate the impact of downsampling depth on fusion performance, we conducted an ablation study focusing on the number of downsampling layers. The results are presented in Table 10. In this study, Setting A and Setting B correspond to network configurations with the maximum downsampling ratios of 1/4 (which corresponds to a three-layer network with two downsampling operations) and 1/16 (corresponding to a five-layer network with four downsampling operations), respectively.

**Table 10:** Quantitative evaluation results of the ablation study on the number of downsampling layers

Setting	VIF	AG	SCD	Q_abf_	SF	EN	SSIM	Average time (ms)
Setting A(3-Layers) (1 1/2 1/4)	1.003	**3.815**	**1.633**	0.692	11.433	6.562	**0.943**	**4.474**
Setting B(5-Layers) (1 1/2 1/4 1/8 1/16)	1.025	3.745	1.628	0.704	11.352	6.554	0.923	8.950
Ours(4-Layers) (1 1/2 1/4 1/8)	**1.055**	3.813	1.633	**0.719**	**11.451**	**6.601**	0.938	6.633

As shown in Table 10, a shallower downsampling scheme (1/4) yields better results in certain metrics such as AG, SCD, and SSIM, but performs relatively poorly in terms of information richness and overall fusion quality. Taking into account fusion accuracy, edge preservation, and computational efficiency, we ultimately adopted a four-layer architecture with a maximum downsampling ratio of 1/8 (denoted as Ours). This configuration achieved either the best or second-best performance across several key metrics, including VIF, Q_abf_, SF, and EN, demonstrating a strong balance between fusion quality and detail preservation.

Moreover, as the downsampling depth increases, the spatial resolution of feature maps in deeper layers is significantly reduced, leading to a gradual loss of edge information at the original scale. When such low-resolution features are directly upsampled and fused with high-resolution representations, the edge information may be further weakened, thereby degrading the overall image quality. Therefore, an appropriate downsampling depth is crucial for maintaining a trade-off between feature abstraction and structural fidelity.

In summary, our approach strikes an effective balance among fusion performance, structural preservation, and computational cost, confirming the rationality and effectiveness of the chosen downsampling strategy.

### Efficiency evaluation

In order to assess the operational efficiency of different algorithms, we selected three metrics for comparison: training parameters, floating-point operations per second (FLOPs), and running time. Additionally, we also provide the average ranks for parameter count and FLOPs, as well as the average rank for running time. Table 11 presents the floating-point operations (FLOPs), training parameters, running time, and their corresponding average ranks for various methods. All our experiments were conducted on an RTX 4090. Specifically, we first evaluated the FLOPs of various methods using resolutions of 256 × 256 and 512 × 512. From Table 11, we observe that some methods have a large number of parameters and higher FLOPs to enhance fusion performance, such as RFN-Nest, SwinFusion, and CDDFuse.

**Table 11:** Comparisons of computational efficiency across different methods. Size rank: The average rank of parameter count and FLOPs. Speed rank: The average rank of runtime. ↓: the lower, the better; (+X↑): rank increased, (−X↓): rank decreased. FLOPs: floating point operations per second

Method	Device/framework	FLOPs (G) ↓		Params (M) ↓	Size rank ↓	Runtime (ms) ↓		Speed rank ↓
		256 × 256	512 × 512			MSRS	M^3^FD	
MST [65]	CPU	-	-	-	-	18.44	60.96	-
DenseFuse [36]	PyTorch	5.82	23.29	0.07	2	**1.07**	**1.98**	**1**(+1↑)
U2Fusion [37]	TensorFlow	86.44	345.75	0.66	7	761.25	1639.27	9(−2↓)
RFN-Nest [39]	PyTorch	111.11	444.42	7.52	9	23.97	31.19	7(+2↑)
SDNet [66]	TensorFlow	8.81	35.25	0.07	3	87.01	233.78	8(−5↓)
SeAFusion [44]	PyTorch	10.88	43.52	0.17	4	1.82	2.62	2(+2↑)
SwinFusion [22]	PyTorch	63.73	254.92	0.93	6	1283.55	3374.52	10(−4↓)
LRRNet [15]	PyTorch	**3.02**	**12.09**	**0.05**	**1**	3.02	3.19	3(−2↓)
CDDFuse [38]	PyTorch	116.85	467.40	1.78	9	17.04	18.44	5(+4↑)
EMMA [67]	PyTorch	8.86	35.45	1.52	5	20.23	24.93	6(−1↓)
RefineFuse(Ours)	PyTorch	12.73	48.11	2.21	7	6.63	9.12	4(+3↑)

Additionally, we selected 361 pairs of images from the MSRS test set and 300 pairs of images from the M^3^FD test set to compare the computational efficiency of different methods. The resolution of MSRS is 640 × 480, while the resolution of M^3^FD is 1024 × 768. It is worth noting that all compared networks are from publicly available source code provided by the authors, and we only calculate the average time taken from the input to the output of the fusion network.

Although LRRNet has the fewest parameters and FLOPs, its internal computations are time-consuming, leading to lower fusion efficiency. In contrast, DenseFuse demonstrates certain advantages in terms of FLOPs, model parameters, and fusion speed. Although our method does not demonstrate a clear advantage in terms of training parameters and FLOPs, it is still satisfactory in terms of efficiency. This is primarily due to our adoption of a multi-scale fusion strategy and an effective downsampling approach to reduce computational load. At the same time, we avoided using complex computational operations during the fusion process, further enhancing fusion efficiency. Overall, our method demonstrates significant advantages in both operational efficiency and fusion quality.

## Conclusion

This paper proposes RefineFuse, a multi-scale feature interaction network for multi-modal image fusion. In RefineFuse, we unify the process of multi-modal fusion tasks in two ways: by preserving texture structures and by appropriately controlling intensities. To balance and exploit local details and global semantic information, we extract multi-scale features from images. We then utilize specific shallow feature interaction modules and deep semantic interaction modules to handle shallow local detail features and deep global semantic features, respectively. Additionally, to alleviate the gap between features at different depths, we designed the feature interaction module (FIM), aiming to leverage deep semantic information to enhance shallow detail features and suppress useless information. The proposed network can produce fused results that have rich texture details and good visual quality. Extensive experiments demonstrate that the proposed fusion network outperforms other methods. Furthermore, experiments in target detection demonstrate the potential of our fusion method for advanced visual tasks.

## Data Availability

The datasets used in this study are publicly available at: MSRS: https://github.com/Linfeng-Tang/MSRS; M^3^FD: https://github.com/JinyuanLiu-CV/TarDAL; TNO: https://figshare.com/articles/dataset/TNO_Image_Fusion_Dataset/1008029; Harvard Medical Dataset: https://www.med.harvard.edu/AANLIB/home.html.

## Abbreviations

AE: auto-encoder
AG: average gradient
CNN: convolutional neural network
CT: computed tomography
DWT: discrete wavelet transform
EN: entropy
FIM: feature interaction block
GAP: global average pooling
GAN: generative adversarial network
IVF: infrared-visible image fusion
LRR: low-rank representation
MIF: medical image fusion
MRI: magnetic resonance imaging
PET: positron emission tomography
PSIM: profound semantic interaction module
SCD: the sum of the correlations of differences
SF: spatial frequency
SFIM: superficial feature interaction module
SPECT: single-photon emission computed tomography
SR: sparse representation
VIF: visual information
